# Lévy Walk in Swarm Models Based on Bayesian and Inverse Bayesian Inference

**DOI:** 10.1016/j.csbj.2020.11.045

**Published:** 2020-12-08

**Authors:** Yukio-Pegio Gunji, Takeshi Kawai, Hisashi Murakami, Takenori Tomaru, Mai Minoura, Shuji Shinohara

**Affiliations:** a Department of Intermedia Art and Science, School of Fundamental Science and Technology, Waseda University, 3-4-1 Ohkubo, Shinjuku-ku, Tokyo, 169-8555, Japan; b Research Center for Advanced Science and Technology, The University of Tokyo Komaba 4-6-1, Meguro-ku, Tokyo, 153-0041, Japan; c Department of Bioengineering, Graduate School of Engineering, The University of Tokyo Hongo 7-3-1, Bunkyo-ku, Tokyo, 113-0033, Japan

**Keywords:** Lévy walk, Swarm Behavior, Bayesian inference, Critical phenomena

## Abstract

While swarming behavior is regarded as a critical phenomenon in phase transition and frequently shows the properties of a critical state such as Lévy walk, a general mechanism to explain the critical property in swarming behavior has not yet been found. Here, we address this problem with a simple swarm model, the Self-Propelled Particle (SPP) model, and propose a way to explain this critical behavior by introducing agents making decisions via the data-hypothesis interaction in Bayesian inference, namely, Bayesian and inverse Bayesian inference (BIB). We compare three SPP models, namely, the simple SPP, the SPP with Bayesian-only inference (BO) and the SPP with BIB models. We show that only the BIB model entails coexisting tornado, splash and translation behaviors, and the Lévy walk pattern.

## Graphical abstract

**Figure:**
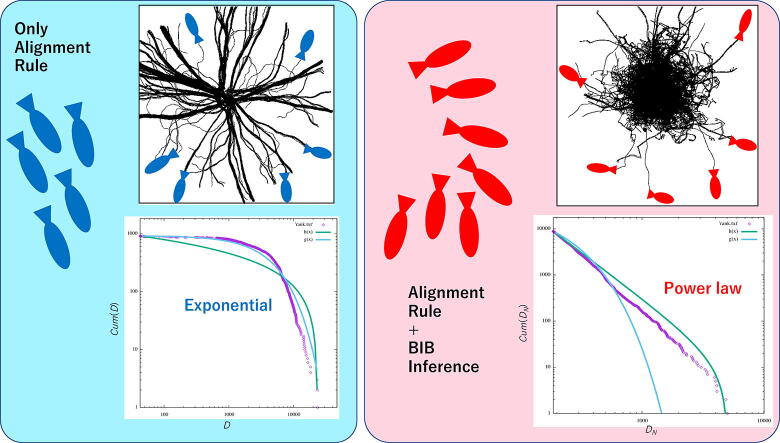

## 1 Introduction

Living systems are adapted to open dynamic environments and can overcome various problems arising in environments [1], [2]. In other words, they can compute what the environments require and can resolve them to some extent. If the problem is well defined, optimization techniques can be applied to the problem. However, the problems living systems face can be ill-defined and can change over time. Notwithstanding these situations, living systems can reach quasi-optimal solutions [3], [4]. While various attempts using bioinspired computing have proposed defining this adaptability [5], [6], [7], [8], [9], [10], the issue of the essential property of adaptability has not yet been found.

A quasi-optimal solution in open environments could be achieved by balancing sticking to a specific well-defined problem with giving up that problem, since open environments could change the problems themselves over time. This can be replaced by balancing highly efficient computation with universal computation [1], [11], balancing exploitation with exploration [12], [13], [14], [15] and balancing specialist with generalist strategies in adaptation [16], [17], [18]. In terms of dynamical systems, balancing the exploitation at an attractor basin with the exploration of wandering various attractors is called the edge of chaos, since staying at an attractor shows oscillation or ordered patterns and wandering to various attractors shows chaotic patterns [19], [20], [21], [22], [23], [24], [25]. It is known that systems tuned at the edge of chaos or at the critical point can have computability, balancing universal computation with highly efficient computation [21], [22], [23], [24], [25]. The critical state is characterized by a power law distribution [26], [27], [28], [29]. What can make a system be tuned to the critical state? Although the idea of self-organizing criticality is one of the candidates [30], [31], [32], no generalized method of tuning at the critical state has been found yet.

Swarming behavior can be a touchstone to determine the core of quasi-optimal solutions in open environments. Recently, many optimization techniques have been developed based on swarming behaviors [33], [34], [35], [36]. A swarm is neither a machine-like order nor just disorder, and a swarm shows a critical state between order and disorder. Since the Self-Propelled Particle (SPP) model is one of the most powerful models for swarms, swarming behavior can be regarded as a critical phenomenon in the phase transition between order and disorder [37], [38]. The SPP model is based only on velocity matching, although other models implement collision avoidance and flock centering [39], [40], [41], [42]. Because the phase transition is controlled by the parameter, the critical state is not self-organizing, and it requires parameter tuning. Indeed, real animal swarms show various properties of the critical state, such as scale-free correlation and Lévy walk, which are characterized by a power law distribution [43], [44], [45], [46], [47], [48], [49]. The problem of what makes a system tuned to the critical state is an open problem.

Here, we show that a critical state balancing exploration (disorder) and exploitation (order) can be obtained through Bayesian inference [50], [51], [52], [53] coupled with the specific interaction between data and hypotheses [54], [55], [56]. We take the SPP model as the basic swarm model and introduce an agent that can infer the state by using Bayesian inference. The question becomes how to obtain the critical behavior of the SPP. The critical behavior can be estimated by a power law distribution of the individual walk pattern, called the Lévy walk. Although there are many approaches to explain the Lévy walk, they are nonsystematic approaches [57], [58], [59]. Swarm approaches based on self-organized criticality [60], [61], [62] have universally failed to explain the Lévy walk [63], [64].

We implement a swarm agent as the decision maker using Bayesian inference coupled with the data-hypothesis interaction. This interaction was previously proposed by us and is called inverse Bayesian inference [54], [55], [56]. Although hypotheses are not altered through Bayesian inference, both the probability and the likelihood of each hypothesis are constantly changed in our model. Normal Bayesian inference does not reveal critical behavior, but our inference system succeeds in explaining the critical behavior or Lévy walk. Real animal swarms, schools and/or flocks exhibit spatial and temporal changes in their collective behaviors, including translation, splash and tornado behaviors. Although detailed parameter tuning is required to reveal these special behaviors in previous swarm models [65], [66], [67], [68], our model, aimed at showing this critical behavior, easily reveals the coexistence of translation, splash and tornado behaviors in a swarm. In the context of the edge of chaos, disturbed patterns and/or disorder are called “chaos” independent of the definition of chaotic dynamics. In our paper, we use the term chaos in this sense, especially disorder.

Since our inference system relying on data-hypothesis interaction easily and ubiquitously shows critical behaviors, the system exhibits universal criticality. Finally, we examine the significance of universal criticality in realizing the optimal design in an open environment for both natural and artificial design purposes.

## 2 Model and Analysis Methods

### 2.1 SPP with Bayesian and inverse Bayesian inference

#### 2.1.1 Simple SPP

The collective behavior in swarms, flocks and schools could contain the various intrinsic dilemma between social norms and individual free decision [69], [70]. One of the simplest models, the Self-Propelled Particle (SPP), implements the social norm by velocity matching of an individual’s neighborhood and their freedom by fluctuation [37], [38]. In our model, the *k*th agent at *t*th time step in a swarm calculates the average velocity in the form of an angle, $\theta_{k}^{t}$,

(1) $$\theta_{k}^{t}=\tan^{-1}\left(\left(\sum_{j}y_{j}^{t}-\sum_{j}y_{j}^{t-1}\right)/\left(\sum_{j}x_{j}^{t}-\sum_{j}x_{j}^{t-1}\right)\right)$$

for any *j*th agent satisfying the following condition:

(2) $${\left(x_{k}^{t-1}-x_{j}^{t-1}\right)}^{2}+{\left(y_{k}^{t-1}-y_{j}^{t-1}\right)}^{2}\leq R^{2}$$

where $\left(x_{k}^{t},y_{k}^{t}\right)$ represents the location of the *k*th agent at the *t*th time step, and R represents the radius of the neighborhood. The (*t*+1)th location of the *k*th agent is determined by:

(3a) $$x_{k}^{t+1}=x_{k}^{t}+V\cos\left(\theta_{k}^{t}\pm rnd\left(\varepsilon \right)+d_{k}^{t}\pi /2\right)$$

(3b) $$y_{k}^{t+1}=y_{k}^{t}+V\sin\left(\theta_{k}^{t}\pm rnd\left(\varepsilon \right)+d_{k}^{t}\pi /2\right)$$

where $rnd\left(\varepsilon \right)$ is the perturbation randomly generated in 0.0≤$rnd\left(\varepsilon \right)$≤*ε*, and $d_{k}^{t}\in D=\left\{0,1,2,3\right\}$ is the angle against the velocity matching inferred through Bayesian and inverse Bayesian inference mentioned later. V is the unit velocity in the form of scalar. It is clear that if $d_{k}^{t}=0$, the *k*th agent accepts the velocity match under a small perturbation, and if $d_{k}^{t}=2$, the *k*th agent escapes from its flock mates in the reverse direction. While *d*=0 reveals ballistic behavior showing exploration, *d*=1, 2 and 3 reveal perturbed behavior showing exploration. Thus, these data reveal variation of exploitation and exploration, and the probability distribution of data constitutes a hypothesis. If $d_{k}^{t}=0$ for any *d* and *t*, then the swarm model is defined as simple SPP or simply SPP.

#### 2.1.2 Bayes Only (BO)

The second Bayes-Only (BO) model is defined by the SPP model equipped with Bayesian inference. Each agent *k* makes $d_{k}^{t}$ by using Bayesian inference. The inference process of the *k*th agent proceeds as follows. In regard to $d_{k}^{t}$ in equation (3), the conditional probability of any hypothesis h∈H={0,1,2,3} under data $d_{k}^{t}$ is expressed as:

(4) $$P_{k}^{t}\left(h|d_{k}^{t}\right)=P_{k}^{t}\left(d_{k}^{t}|h\right)P_{k}^{t}\left(h\right)/\sum_{h}P_{k}^{t}\left(d_{k}^{t}|h\right)P_{k}^{t}\left(h\right)$$

where $P_{k}^{t}\left(h|d\right)$ is the conditional probability of *h* under *d* used by the *k*th agent at the *t*th step, $P_{k}^{t}\left(d|h\right)$is the conditional probability of *d* under *h*, called the likelihood, and $P_{k}^{t}\left(h\right)$is the probability of *h*. The hypothesis $h_{k}^{t}$ is a hypothesis that individual *k* makes at time *t* about the state of the swarm. On the other hand, $d_{k}^{t}$ is the state of individual *k* at time *t*. Since $P_{k}^{t}\left(d\right)=\sum_{h}P_{k}^{t}\left(d|h\right)P_{k}^{t}\left(h\right)$, it is clear that equation (4) reveals the Bayes formula such that $P_{k}^{t}\left(d|h\right)P_{k}^{t}\left(h\right)=P_{k}^{t}\left(h|d\right)P_{k}^{t}\left(d\right)$. In Bayesian inference, the probability of any *h* is replaced by the conditional probability of *h* under $d_{k}^{t}$, which is expressed by:

(5) $$P_{k}^{t+1}\left(h\right)=P_{k}^{t}\left(h|d_{k}^{t}\right)$$

Initially, for any *d* and *h*, $P_{k}^{1}\left(d|h\right)$, $P_{k}^{1}\left(h\right)$ and $d_{k}^{1}$ are given. Then for any *t* and any *h,* $P_{k}^{t}\left(h|d_{k}^{t}\right)$ is determined by equation (4), and then $P_{k}^{t+1}\left(h\right)$ is determined by $P_{k}^{t+1}\left(h\right)=P_{k}^{t}\left(h|d_{k}^{t}\right)$, equation (5). With the use of the updated $P_{k}^{t+1}\left(h\right)$ as equation (5), the hypothesis with the highest probability can be chosen dependent on its probability. This implies that $h_{k}^{t+1}\in H$ satisfying the following:

(6) $$P_{k}^{t+1}\left(h_{k}^{t+1}\right)\geq P_{k}^{t+1}\left(h\right)$$

for any h∈H can be chosen based on the probability. The cumulative probability of the hypothesis such that:

(7) $${\mathrm{CP}}_{k}^{t+1}=\sum_{h}P_{k}^{t+1}\left(h\right)$$

is defined, and random variable 0.0≤r≤1.0 is then updated. The updating of $h_{k}^{t+1}$ is determined by:

(8) $$h_{k}^{t+1}=\min\left\{h\in H|r\leq {\mathrm{CP}}_{k}^{t+1}\left(h\right)\right\}$$

After $h_{k}^{t+1}$ is determined, data $d_{k}^{t+1}$satisfying:

(9) $$P_{k}^{t+1}\left(d_{k}^{t+1}|h_{k}^{t+1}\right)\geq P_{k}^{t+1}\left(d|h_{k}^{t+1}\right)$$

for any d∈D can be chosen with the corresponding probability. The procedure based on the probability is the same as that in equations (7-9). The cumulative probability conforming to:

(10) $${\mathrm{DP}}_{k}^{t+1}\left(d|h_{k}^{t+1}\right)=\sum_{d}P_{k}^{t+1}\left(d|h_{k}^{t+1}\right)$$

is defined, and random variable 0.0≤p≤1.0 is then updated. The updating of $d_{k}^{t+1}$ is determined by

(11) $$d_{k}^{t+1}=min\left\{d\in D|p\leq {\mathrm{DP}}_{k}^{t+1}\left(d|h_{k}^{t+1}\right)\right\}$$

Bayesian inference consists of the above procedure equations (4)-(11).

As mentioned before, in Bayesian inference, a set of hypotheses is not changed through the inference process. Since a hypothesis is defined by its likelihood such as P(d|h), the distribution of P(d|h) is not altered in Bayesian inference. In contrast, here, we introduce the interaction between data and hypotheses below. If the swarm model is implemented by equations (1)-(11) and $P_{k}^{t}\left(d|h\right)$ is invariant over time, we call the model an SPP with BO model, or simply a BO model.

##### 2.1.2.1 Bayesian and Inverse Bayesian Inference (BIB)

The third swarm model based on the SPP is called the SPP with Bayesian and Inverse Bayesian Inference (BIB) model, or simply a BIB model. In addition to equations (1)-(11), the following equations (12)-(17) are also implemented in the BIB model.

Given a set of data, such as $\mathrm{Dat}=\{\left(d^{1},1\right),\left(d^{2},2\right),\cdots ,\left(d^{m},m\right)\}$ for $d^{i}\in D$, one can obtain the probability of *d* as the normalized frequency:

(12) $$P\left(d\right)=\#\left\{\left(d^{i},i\right)\in Dat\right|d^{i}=d\}/m$$

where *#S* for set *S* is the number of elements in *S*, and #Dat=m (window size). For instance, given {(0, 1), (0, 2), (2, 3), (1, 4)}, *P*(0) = #{(0, 1), (0, 2)}/4=0.5, *P*(1) = #{(1, 4)}/4=0.25, *P*(2) = #{(2, 3)}/4=0.25 and *P*(3) = #{}/4 =0. In our model, for a set of time series, given $\mathrm{Dat}=\{\left(d_{k}^{t-m+1},1\right),\cdots ,\left(d_{k}^{t-1},m-1\right),\left(d_{k}^{t},m\right)\}$, the probability of *d* for the *k*th agent is defined by:

(13) $$P_{k}^{t}\left(d\right)=\#\left\{\left(d_{k}^{t-w},m-w\right)\in Dat|d_{k}^{t-w}=d\right\}/m$$

The interaction between data and hypotheses or the inverse Bayesian inference is defined by:

(14) $$P_{k}^{t+1}\left(d|f_{k}^{t+1}\right)=P_{k}^{t}\left(d\right)$$

where $f_{k}^{t+1}\in H$satisfies:

(15) $$P_{k}^{t+1}\left(h\right)\geq P_{k}^{t+1}\left(f_{k}^{t+1}\right)$$

for any h∈H, which can be chosen based on the probability. With the use of the cumulative probability defined as:

(16) $${\mathrm{EP}}_{k}^{t+1}\left(h\right)=\sum_{h}\left(1-P_{k}^{t+1}\left(h\right)\right)$$

$f_{k}^{t+1}$is determined by the probability, for any given random variable 0.0≤r≤3.0, and $f_{k}^{t+1}$ is obtained by:

(17) $$f_{k}^{t+1}=min\left\{h\in H|r\leq {\mathrm{EP}}_{k}^{t+1}\left(h\right)\right\}$$

For instance, if $P_{k}^{t+1}\left(0\right)=0.2$, $P_{k}^{t+1}\left(1\right)=0.1$, $P_{k}^{t+1}\left(2\right)=0.4$ and $P_{k}^{t+1}\left(3\right)=0.3$, then ${\mathrm{EP}}_{k}^{t+1}\left(0\right)=0.8$, ${\mathrm{EP}}_{k}^{t+1}\left(1\right)=1.7$, ${\mathrm{EP}}_{k}^{t+1}\left(2\right)=2.3$, and ${\mathrm{EP}}_{k}^{t+1}\left(3\right)=3.0$. Thus, it is the most convenient for *r* to reach the region between ${\mathrm{EP}}_{k}^{t+1}\left(0\right)$ and ${\mathrm{EP}}_{k}^{t+1}\left(1\right)$. This results in $f_{k}^{t+1}=1$.

It is evident that $P_{k}^{t+1}\left(h\right)=P_{k}^{t}\left(h|d_{k}^{t}\right)$ in equation (5) is symmetric to $P_{k}^{t+1}\left(d|f_{k}^{t+1}\right)=P_{k}^{t}\left(d\right)$ in equation (14), which is why the procedure of equation (14) is called inverse Bayesian inference. On the one hand, Bayesian inference contracts the condition of an event (hypothesis) by replacing the probability of $P_{k}^{t+1}\left(h\right)$ with the conditional probability of $P_{k}^{t}\left(h|d_{k}^{t}\right)$. On the other hand, inverse Bayesian inference extends the condition of an event (data) by replacing $P_{k}^{t+1}\left(d|f_{k}^{t+1}\right)$ with $P_{k}^{t}\left(d\right)$. Thus, Bayesian inference balances contraction (exploitation) with extension (exploration). This is considered in a later section and compared to the mechanism of self-organized criticality.

**Figure 1:**
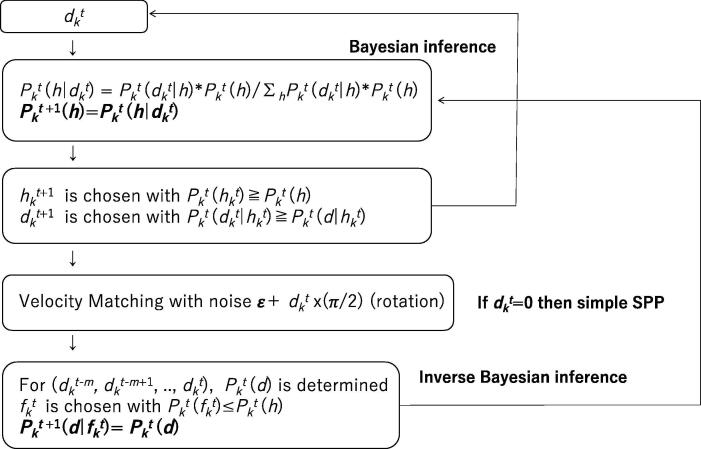
Schematic diagram of the algorithm for the SPP with Bayesian and inverse Bayesian inference. In all simulation studies in this paper, *h* and *d* are chosen from {0, 1, 2, 3}.

In our simulation studies, the program for the SPP in which Bayesian and inverse Bayesian inference (BIB) are implemented is shown in Figure 1. In the program, if $d_{k}^{t}=0$ for any *t*, the program simulates the simple SPP under which each agent obeys the velocity matching expressed by equations (1)-(3) without any inference. If certain sourced codes implying equations (12)-(17) are commented out, the program simulates the SPP containing only Bayesian inference. This implies the likelihood of hypotheses $P_{k}^{t}\left(d|h\right)=P_{k}^{t-1}\left(d|h\right)$ for any *d* and *h*. It is easy to compare the simple SPP, the SPP with only Bayesian inference (BO), and SPP with BIB.

The SPP part of our model contains various parameters, the radius of the neighborhood, R, the unit velocity of each agent, V, and the perturbation (noise) for the velocity matching, *ε* (radian). Throughout all simulation studies, we set R=20.0 and V =5.0 if there is no description. The perturbation *ε* varies and corresponds to the phase parameter controlling the phase shift.

### 2.2 Step length distribution for the power law analysis

To estimate the critical behavior in the model swarm, the distribution of the step length is measured for the simulation studies. In research on animal foraging, the step length distribution for various animals has been measured and analyzed with respect to the power law distribution [71], [72], [73], since animal foraging always encounters exploitation and exploration dilemmas. If animals implement the exploitation strategy and consume food resources in a closed environment, the walking patterns reveal random walks. In contrast, if animals implement the exploration strategy to search for other food resources and leave their previous environments, the animals reveal ballistic walking trajectories, which could realize efficient searches for unknown resources. If the step length is defined by the distance between two bending points, the ballistic walk implies a very long step length, while the random walk implies a short step length normally distributed around the mean step length. Animals could balance exploitation with exploration, and the walk pattern would reveal a random walk pattern with a long tail, which is characterized by a power law distribution of the step length. If the exponent of the power law distribution ranges from 1.0 and 3.0, the walk pattern is called the Lévy walk. It is known that the foraging patterns of animals frequently indicate the Lévy walk [43], [44], [45], [46], [47].

Here, we define the step length by the following. Since the location of an agent is moved in a stepwise fashion, we define the bending angle *α* in the agent’s walk as:

(18) $$\alpha^{t}=\cos^{-1}\left(\left(\left(x_{k}^{t}-x_{k}^{t-1}\right)\left(x_{k}^{t-1}-x_{k}^{t-2}\right)+\left(y_{k}^{t}-y_{k}^{t-1}\right)\left(y_{k}^{t-1}-y_{k}^{t-2}\right)\right)/V^{2}\right)$$

where$\left(x_{k}^{t},y_{k}^{t}\right)$ is the location of the *k*th agent at the *t*th time step. In the walking pattern of agents, if $\alpha^{t}>\alpha_{max}$, one step walk is then terminated, and the distance, *D*, between the previous bending point $\left(X_{k},Y_{k}\right)$ and the new bending point $\left(x_{k}^{t-1},y_{k}^{t-1}\right)$ is obtained by:

(19) $$D={\left({\left(X_{k}-x_{k}^{t-1}\right)}^{2}+{\left(Y_{k}-y_{k}^{t-1}\right)}^{2}\right)}^{1/2}$$

This is consistent with the step length as the intermittent interval length [74]. After calculating the step length, the previous bending point is updated by:

(20) $$X_{k}=x_{k}^{t-1},Y_{k}=y_{k}^{t-1}$$

The power law distribution of the step length is estimated with respect to the frequency distribution of *D*. In our analysis in this paper, we define $\alpha_{max}=2\pi /9$.

### 2.3 Analysis of the translation, splash and tornado behaviors

As mentioned before, the critical behavior in artificial systems indicates universal and efficient computation. This behavior is characterized by the complex mixing of contraction and extension of information. In cellular automata, the critical behavior consists of locally stable oscillatory or fixed patterns and chaotic wave patterns propagating from one local site to another [22], [23]. Natural and real animal groups also exhibit these behaviors. Locally fixed patterns and locally oscillating patterns are compared to translating movements (or schooling) and tornado (or massive tornado) patterns, respectively [65], [67], [68]. The chaotic propagating waves are compared to the splash patterns of animal groups. While the coexistence of translation, splash and tornado behaviors seems to be the attribute specific to the critical behavior, previous swarm models have never revealed the coexistence of these behaviors. Indeed, splash and tornado patterns require fine tuning of the parameters and initial conditions.

Therefore, it is very important to estimate the coexistence of translation, splash and tornado patterns in swarms in terms of the critical behavior. Here, we define the index for the tornado pattern in a swarm by the number of agents satisfying the following:

(21) $$\alpha_{tor}^{\text{'}}>\sum \alpha^{t}>\alpha_{tor}$$

Inequality (21) implies that the average bending angle of the agent’s walk is so large that the agent rotates around a point. Since the splash pattern implies that agents are radially dispersed, the index for the splash pattern is defined by the number of agents satisfying the following:

(22) $$r_{\min}^{t}-r_{\min}^{t-T}>r_{spl}$$

where $r_{\min}^{t}$ denotes the distance between the *k*th agent and its nearest neighbor, and T represents a constant time interval. Thus, inequality (22) implies that the nearest neighbors come away. In contrast, since the translation pattern of a swarm implies that agents move without changing their moving direction, the index for the translation pattern is defined by the number of agents satisfying the following:

(23) $$\sum \alpha^{t}<\alpha_{trans}$$

**Figure 2:**
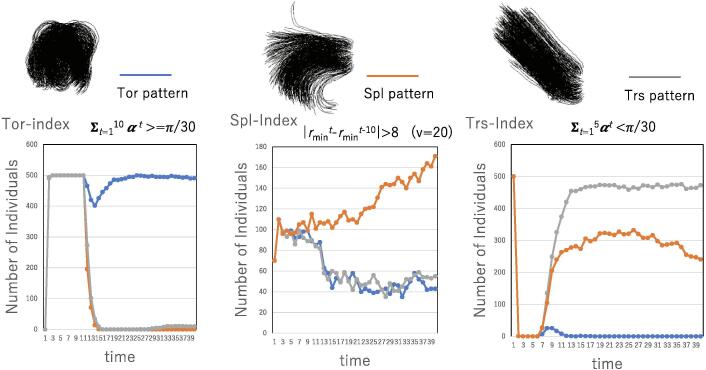
Indexes for the tornado (Tor) pattern, splash (Spl) pattern and translation (Trs) pattern in swarms. Given the typical tornado, splash and translation patterns in a swarm model (top diagrams), the number of agents satisfying the corresponding indexes is plotted over time. The blue, orange and gray lines represent the tornado, splash and translation patterns (bottom), respectively.

Figure 2 (top) shows snapshots of the three patterns of swarm behaviors. The left snapshot depicts the tornado patterns in which all 500 agents rotate. The middle snapshot shows the splash patterns in which 500 agents are dispersed from the right to the left. The right snapshot shows the translation patterns from below to above. These snapshots are simulated by the SPP-based model implemented to reveal each pattern. The bottom graph shows the number of agents satisfying conditions (21), (22), (23) over time for each swarm pattern. In the left graph, first, all agents match their velocity resulting in the translation pattern, after which all agents rotate. Thus, soon after starting to rotate, the number of agents satisfying condition (21) (i.e., the tornado index) increases. In the middle graph, the number of agents satisfying condition (22) (i.e., the splash index) gradually increases. In the right graph, the number of agents satisfying condition (23) (i.e., the translation index) increases, while there are some agents exhibiting the splash pattern due to the perturbation in the SPP.

Note that the three indexes are not complementary to each other, and one agent can therefore satisfy multiple conditions. Notwithstanding this ambiguity, Figure 2 shows that the three indexes are suitable factors to estimate the behavioral components of the splash, tornado and translation patterns.

## 3 Results

All the experiments were conducted on a PC with an Intel Core 17 processor running at 2.6GHz, and hard drive of 16 Gbytes. Our implementation was compiled using gcc (4.2.1).

### 3.1 Critical behavior consisting of splash, tornado and translation patterns

In this paper, we use the term criticality both in the phenomenological sense and in the strict sense. Criticality in the strict sense is defined here by the phenomena characterized by a power law distribution. In contrast, criticality in the phenomenological sense implies the midpoint of the transition between order and disorder in a broad sense. This phase transition is expressed as the phase transition between the ordered swarm showing translation and/or tornado behavior and the disordered swarm showing splash behavior. While tornado behavior is a typical exploitation behavior, splash and translation behaviors are typical exploration behaviors, in another sense. The coexistence of tornado, splash and translation behaviors implies criticality in the phenomenological sense. Therefore, the question arises whether criticality in the phenomenological sense entails criticality in the strict sense.

**Figure 3:**
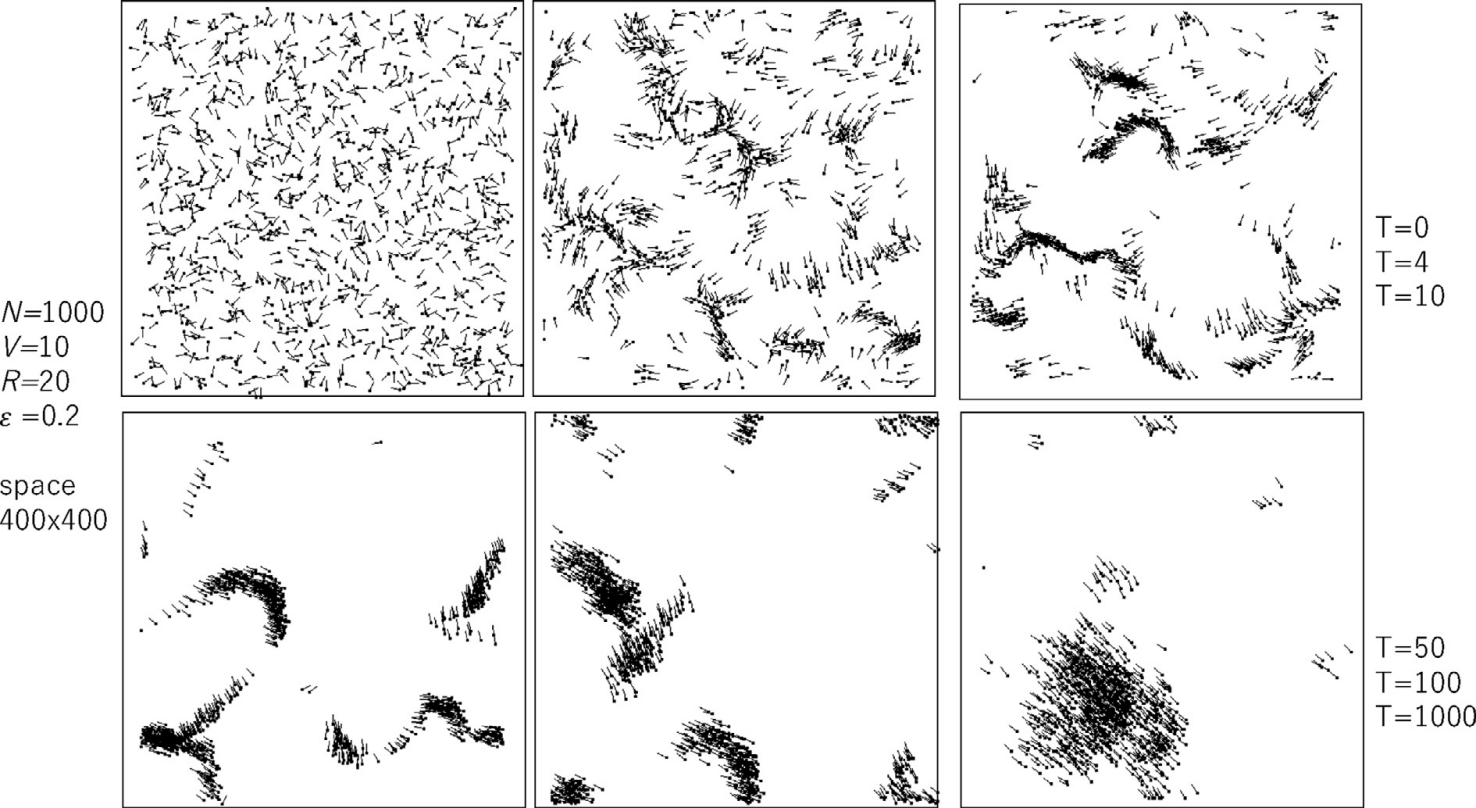
Snapshots of SPP model development over time. The time proceeds from the top left (initial condition) to the top right and then from the bottom left to the bottom right. The boundary condition is the wrapped condition.

Figure 3 shows the time development of the SPP model. Each diagram shows a snapshot of the swarm consisting of 1000 agents, where the *k*th agent is represented by the line connecting its location $\left(x_{k}^{t-1},y_{k}^{t-1}\right)$ with $\left(x_{k}^{t},y_{k}^{t}\right)$. Although the SPP does not implement flock centering (the force to approach the denser swarm) but only velocity matching, the agents are gradually concentrated due to the periodic boundary condition (i.e., the left margin of the square space is connected to the right margin, and the top of it is connected to its bottom). This results in a dense translation pattern, as shown in the right bottom snapshot. Typical time development is stored as Video_simple_SPP_1.

It is known that the SPP exhibits a phase transition expressed as polarization (degree of velocity matching) with respect to normalized perturbations. The original SPP, which implements only velocity matching, could reveal neither tornado nor splash patterns. If the SPP is coupled with both a self-propelled force and friction, a specific ratio of the force and friction could lead to self-organizing tornado patterns [67], [68]. Not only the SPP but also other swarm models could entail tornado patterns under attraction and repulsion balancing [39], [65], [66]. Tornado patterns require fine tuning of the parameter setting. Although the splash pattern is frequently observed in real bird flocks and fish schools, it has been examined with respect to the prey-predator scheme in simulation studies. Since the prey-predator scheme is implemented by balancing attraction and repulsion, not only the prey-predator scheme but also the general swarm model coupled with attraction and repulsion could reveal splash patterns. However, fine tuning of the parameters is required.

Our SPP coupled with Bayesian inference contains both decisions consistent with and contradictory to velocity matching. Thus, it could be similar to the balancing of the self-propelled force and friction and the balancing of attraction and repulsion. This suggests that the SPP coupled with only Bayesian inference (i.e., BO model) could reveal tornado and splash patterns.

**Figure 4:**
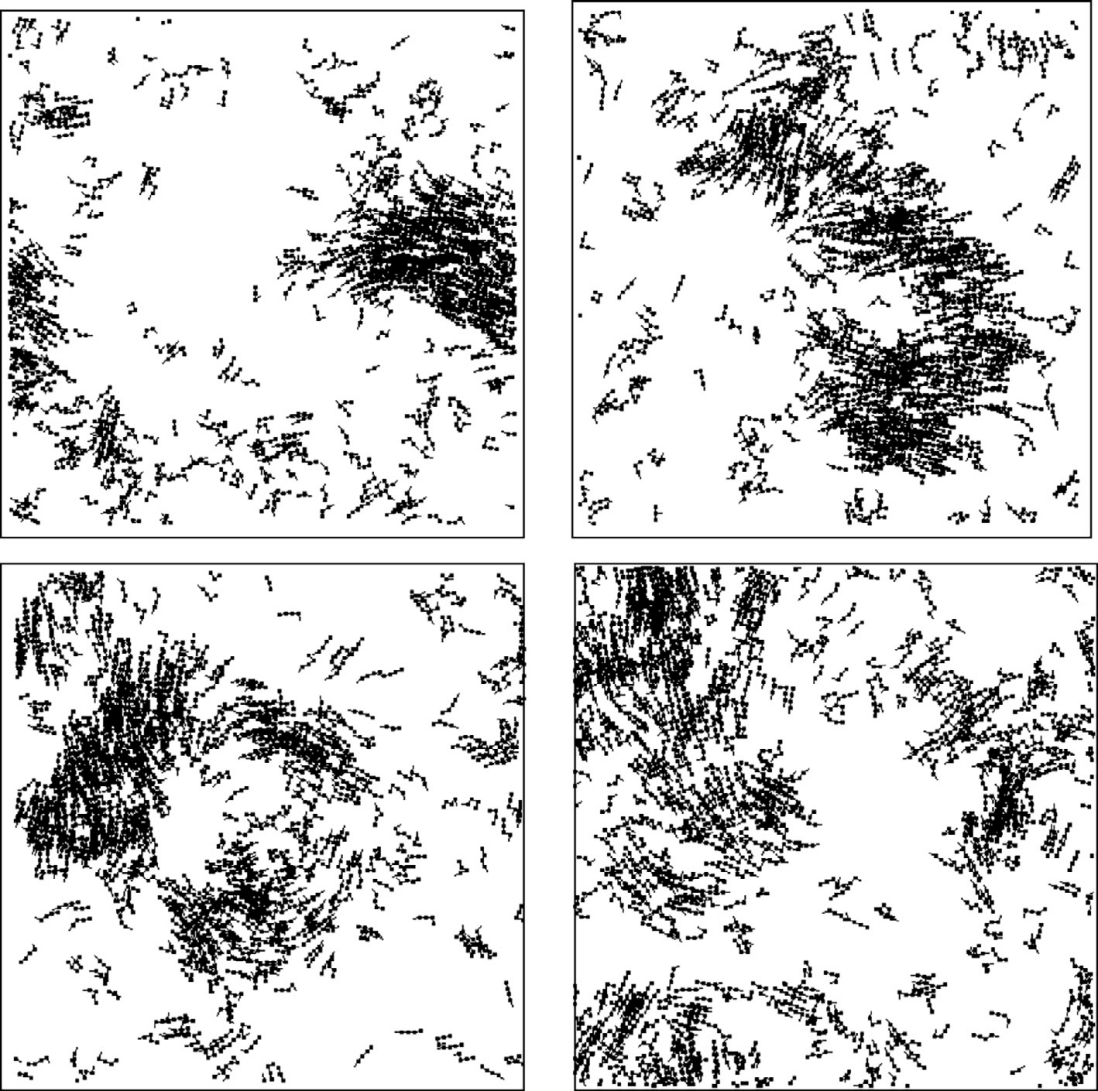
Snapshots of the SPP coupled with Bayesian inference (BO model). The trajectory of each agent consists of four successive stepwise positions. The boundary condition is the wrapped condition, for R=20.0 V =5.0, *ε*=0.001, and N=1000.

Figure 4 shows short trajectories of the SPP coupled only with Bayesian inference. Since the degree of obeying the velocity matching is determined by Bayesian inference, this behavior depends on the likelihood of the hypotheses, where $P_{k}\left(d|h\right)$ is invariant throughout time for each *k*th agent. In our model, for any *k,* $P_{k}\left(d|h\right)=0.7$ if d=h; otherwise, $P_{k}\left(d|h\right)=0.1$. Through time development, $P_{k}\left(d|h\right)$ is not altered, and initially, $P_{k}\left(h\right)=0.25$ for any h∈H.

While each agent affects $P_{k}\left(h\right)$ dependent on its previous data, $d_{k}^{t}$, no behaviors characterized by critical behavior are observed. Each agent frequently changes its decision to either obey the velocity matching or not, and various rotations against the mean velocity are then mutually canceled. This leads to a fluctuating moving swarm, which sometimes indicates a large swarm and is sometimes divided into small parts. Dispersing and gathering patterns are perpetually iterated and indicate a complex fluctuating behavior, although they never show explicit splash and/or tornado patterns. If $P_{k}\left(d|h\right)$ is randomly given and remains invariant over time, while the swarm rarely shows a tornado. However, in these cases, the realized tornado is perpetually sustained and not broken. This case seems to be achieved by the random setting of$P_{k}\left(d|h\right)$ and could be compared to the fine parameter tuning by chance. Typical time development of SPP only with Bayesian inference is stored as Video_BO_1.

**Figure 5:**
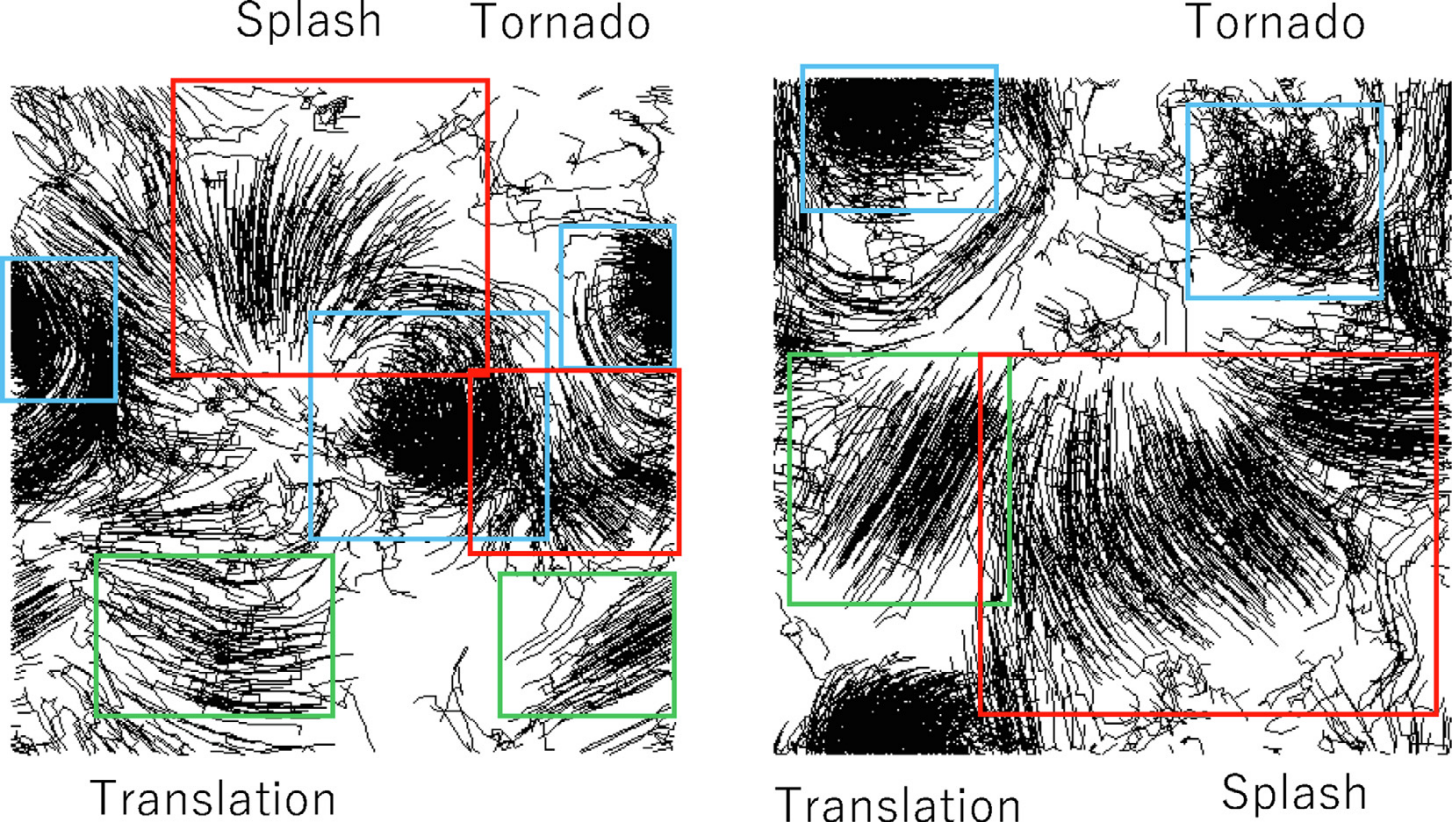
Snapshots of the SPP coupled with Bayesian and inverse Bayesian inference. The trajectory of each agent consists of 20 successive stepwise positions. The boundary condition is the wrapped condition, for R=20.0 V =5.0, *ε*=0.001, and N=1000. The translation, splash and tornado patterns are marked with green, blue and red squares, respectively.

Figure 5 shows a pair of snapshots of the SPP coupled with Bayesian and inverse Bayesian inference (i.e., BIB model). Typical time development of SPP with Bayesian and inverse Bayesian inference is stored as Video_BIB_1. It is evident that there are translation, splash and tornado patterns. Due to the coexistence of translation, splash and tornado behaviors, the swarm is perpetually dispersed and gathered. Since the initial likelihood of$P_{k}^{t}\left(d|h\right)$ is rapidly replaced by another one and is perpetually altered, complex patterns consisting of translation, splash and tornado behaviors occur independent of the initial condition of the distribution of agents and the initial condition of $P_{k}^{t}\left(d|h\right)$. Compared to the SPP with only Bayesian inference (i.e., BO model), there are distinct patterns of locally stable information processing manifested as a tornado pattern and of information transmission manifested as splash and translation patterns. In the BO, information processing and transmission are mixed and averaged, which involves perturbed and fuzzy information processing. Since the BO seems neither chaotic nor exhibits definite information processing, it cannot be used for universal and efficient information processing.

**Figure 6:**
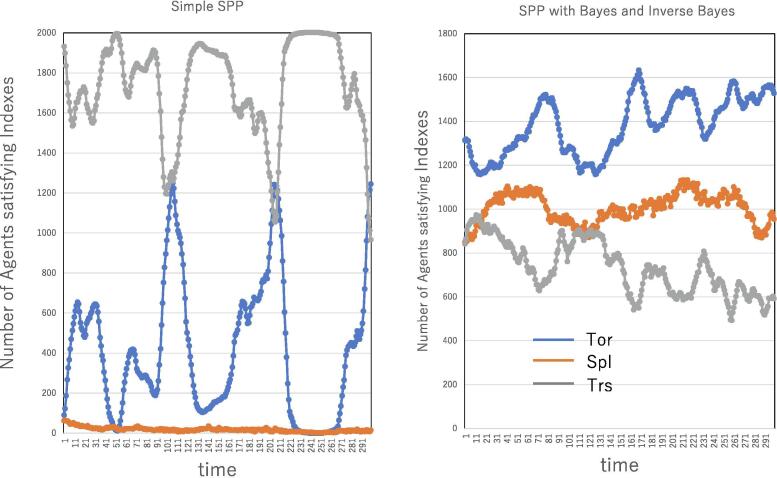
Tornado, splash, and translation analysis for the simple SPP (left) and the SPP coupled with Bayesian and inverse Bayesian inference (right). The number of individuals (agents) satisfying the tornado index (blue), splash index (orange) and translation index (gray) are plotted over time. R=20.0 V =5.0, *ε*=0.1, N=2000, $\alpha_{\mathrm{tor}}=\alpha_{\mathrm{trans}}=\pi /30$, $\alpha_{tor}^{\text{'}}=\pi /10$ and $r_{spl}=8.0$.

The indexes of the tornado, splash and translation patterns are estimated here for the behavior of the swarm. Figure 6 shows a comparison of the indexes in the SPP and the BIB. The indexes of the tornado, splash and translation patterns are plotted over time. In the SPP, there is no indication of the splash pattern, and the swarm shows a mixture of translation and tornado patterns due to the perturbation, while the major phenomenon is translation. After a very large swarm is generated, translation occurs due to velocity matching. Thereafter, due to the perturbation, the swarm is divided into various parts, and one swarm is again formed. In the dividing process, a small moving population is split to maintain a dense population. Therefore, swarm deformation does not influence the index of the splash pattern but affects that of the tornado pattern.

In contrast, the BIB is characterized by the coexistence of tornado, splash and translation behaviors. As mentioned above, since the indexes of the tornado, splash and translation patterns are not independent of each other, they are mixed, and the perturbed splash and/or perturbed tornado pattern can thus be estimated by both the tornado and splash indexes. As shown in Figure 6 (right), all indexes are high in the swarm of the BIB.

**Figure 7:**
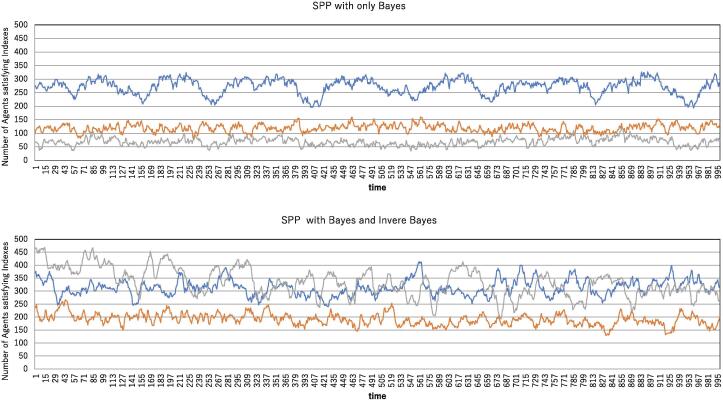
Tornado, splash, and translation analysis for the SPP with only Bayesian inference (BO) and for the SPP coupled with Bayesian and inverse Bayesian inference (BIB). The number of agents satisfying the tornado index (blue), splash index (orange) and translation index (gray) are plotted over time. R=20.0 V =5.0, *ε*=0.001, N=1000,$\alpha_{\mathrm{tor}}=\alpha_{\mathrm{trans}}=\pi /30$, $\alpha_{tor}^{\text{'}}=\pi /10$ and $r_{spl}=8.0$.

Figure 7 shows a comparison of the BO and the BIB with respect to the tornado, splash and translation indexes. Both simulation results are obtained under low-fluctuation conditions. Although both simulations are performed for 1000 agents, the summation of the agents satisfying the three indexes is much smaller than 1000 for the BO. This implies that there are many agents satisfying neither tornado nor translation indexes whose average turn angles are over π/10. Since the BO allows agents to neglect velocity matching, agents can turn at a sharp angle. If this fluctuating behavior resulting from Bayesian inference is simply mixed with the agents obeying velocity matching, it is considered that there are many agents that can turn at an angle other than π/10. In contrast, the BIB reveals that both the tornado and translation indexes are satisfied by a high proportion of agents and indicates that the agents satisfying the three indexes are complementary with each other. This implies that splash, translation and tornado behaviors coexist in the swarm and that these three behaviors continuously connect with each other as if the information originating from the tornado pattern is effectively transmitted to other places by the splash and translation patterns.

**Figure 8:**
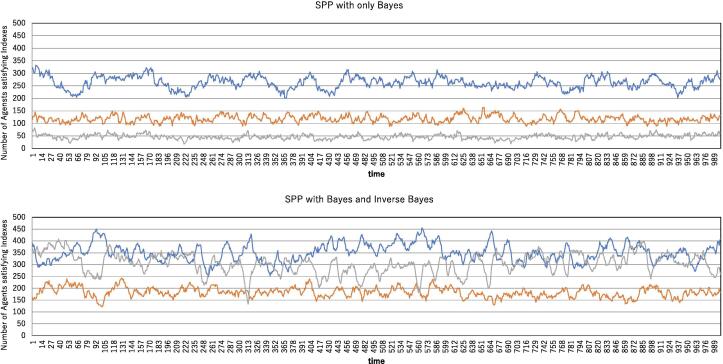
Tornado, splash, and translation analysis for the SPP with only Bayesian inference (BO) and the SPP coupled with Bayesian and inverse Bayesian inference (BIB). The number of agents satisfying the tornado index (blue), splash index (orange) and translation index (gray) are plotted over time. All parameters except for *ε* are the same as those in Figure 7, and *ε*=0.2.

The simulation results in Figure 7 are obtained under the condition of a small perturbation. The question arises whether the difference between the two kinds of SPPs, BO and BIB, results from this small perturbation. Therefore, we simulated under the condition of a large perturbation. Figure 8 shows the simulation results under a perturbation *ε* of 0.2. The trend depicted in Figure 7 is also observed in Figure 8. This reveals that adequate combinations of local information processing and information transmission are realized by the BIB, independent of the perturbation magnitude.

**Figure 9:**
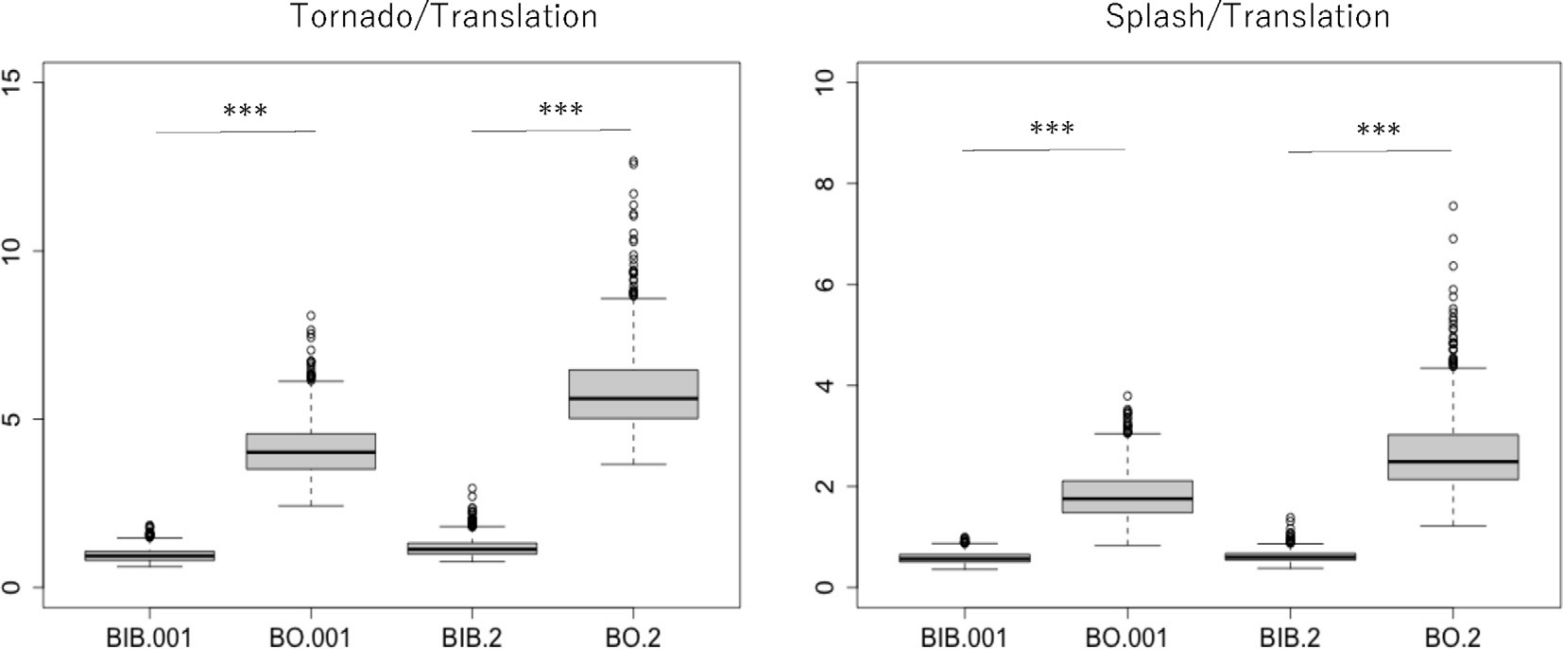
Comparison between the BIB and BO models with respect to the ratio of individuals showing tornado behavior to those showing translation behavior (left) and the ratio of individuals showing splash behavior to those showing translation behavior (right). The number .001 represents the condition *ε*=0.001, and .2 represents *ε*=0.2.

We conducted a statistical test to assess the difference between the BIB and BO models with respect to the tornado-translation index and the splash-translation index (Figure 9). The normality was tested by the Shapiro-Wilk normality test, and normality was rejected (p < 0.001, all are smaller than 7.443e-14). The difference between the mean value of the ratio in the BIB model and that in the BO model was checked by the Wilcoxon rank sum test, and the results showed a significant difference between them under both conditions, small perturbation and large perturbation (p < 0.001, all are smaller than 2.2e-16). The statistical data for the test are shown in Table 1.

**Table 1:** Statistical data for the ratio of individuals showing tornado behavior to those showing translation behavior (left) and the ratio of individuals showing splash behavior to those showing translation behavior (right).

	BIB_.001	BO_.001	BIB_.2	BO_.2			BIB_.001	BO_.001	BIB_.2	BO_.2
Min.	0.6212	2.421	0.7615	3.659		Min.	0.3571	0.8288	0.3799	1.216
1st Qu	0.8022	3.517	0.9913	5.019		1st Qu	0.5058	1.4817	0.5446	2.138
Median	0.9383	4.017	1.1335	5.614		Median	0.5697	1.7571	0.6	2.491
Mean	0.9684	4.112	1.1834	5.858		Mean	0.5856	1.8284	0.613	2.679
3rd Qu	1.0764	4.566	1.3178	6.462		3rd Qu	0.6537	2.111	0.6758	3.027
Max.	1.8447	8.077	2.9474	12.667		Max.	0.9909	3.7949	1.3835	7.556

The tendencies found in Figure 9 are general trends in the BIB and BO models. While the behaviors in the BO model are basically perturbed translation, which is revealed by the tornado index, the behaviors in the BIB model are basically characterized by the coexistence of tornado, splash and translation behaviors. Thus, the tornado index normalized by the translation index and the splash index normalized by the translation index in the BIB model are smaller than those in the BO model.

In addition, we simulated the special case of the SPP model, in which every individual has a different radius. In that case, individuals converge to a large swarm controlled by the smallest radius. Thus, it can be concluded that individuals with heterogeneous radii never generate the coexistence of translation, tornado and splash behaviors.

### 3.2 Power law distribution in the SPP with Bayesian and inverse Bayesian inference

Next, we determine whether the SPP with Bayesian and inverse Bayesian inference (BIB model) shows critical behavior in a term of power law distribution. In estimating the translation, splash and tornado behaviors, the periodic boundary condition is defined as to not disperse the swarm. In estimating the power law distribution of the step length, 1000 agents are initially located in a small central area in an open space, and the walking pattern of any freely moving agents is estimated.

**Figure 10:**
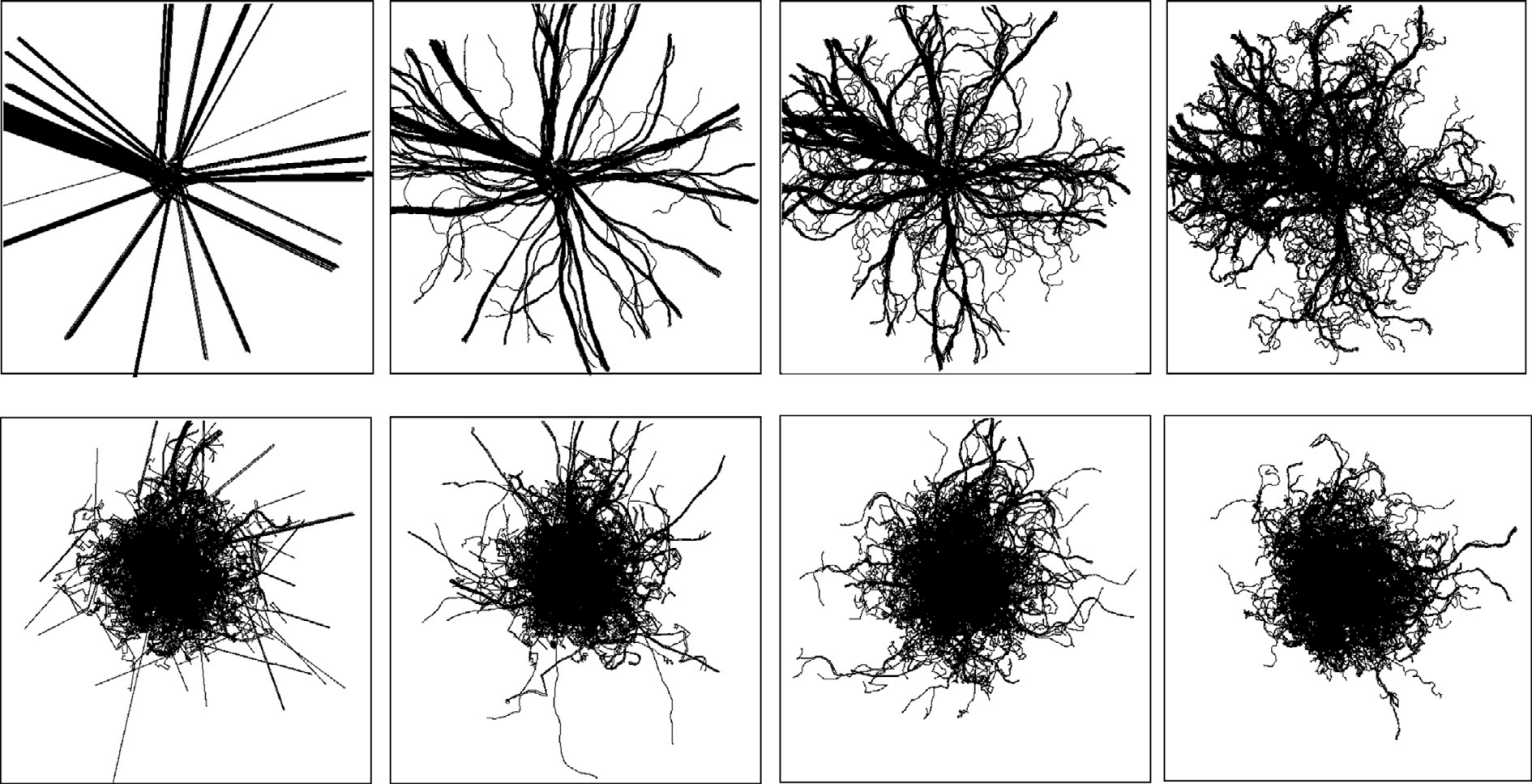
Trajectories from a central area of the SPP (above) and of the BIB (below), for *t*=500, R=20.0 V =5.0, and N=1000. The perturbation *ε* is 0.001, 0.1, 0.2 and 0.4 from left to right.

Figure 10 shows the trajectories of the simple SPP (above) and the BIB swarm. Each column shows the conditions of *ε*=0.001, 0.1, 0.2 and 0.4. The SPP model with *ε*=0.001 (above left) shows ballistic trajectories in finite time since little perturbation influences the trajectories. Since the simulations were run for *T*=10000, most agents remained in a central area, and alignm ent interactions also contributed to the step length distribution under a large perturbation. The results of the power law distributions are the same as those under periodic boundary conditions. The more perturbations there are, the more rotations occur indicating plant-root-like networks. In contrast to the SPP, the BIB swarm reveals similar trajectory patterns independent of the extent of the perturbation. While there are no trajectories of the BO swarm in Figure 10, the apparent patterns are similar to those generated by the BIB, where there are no ballistic trajectories.

**Figure 11:**
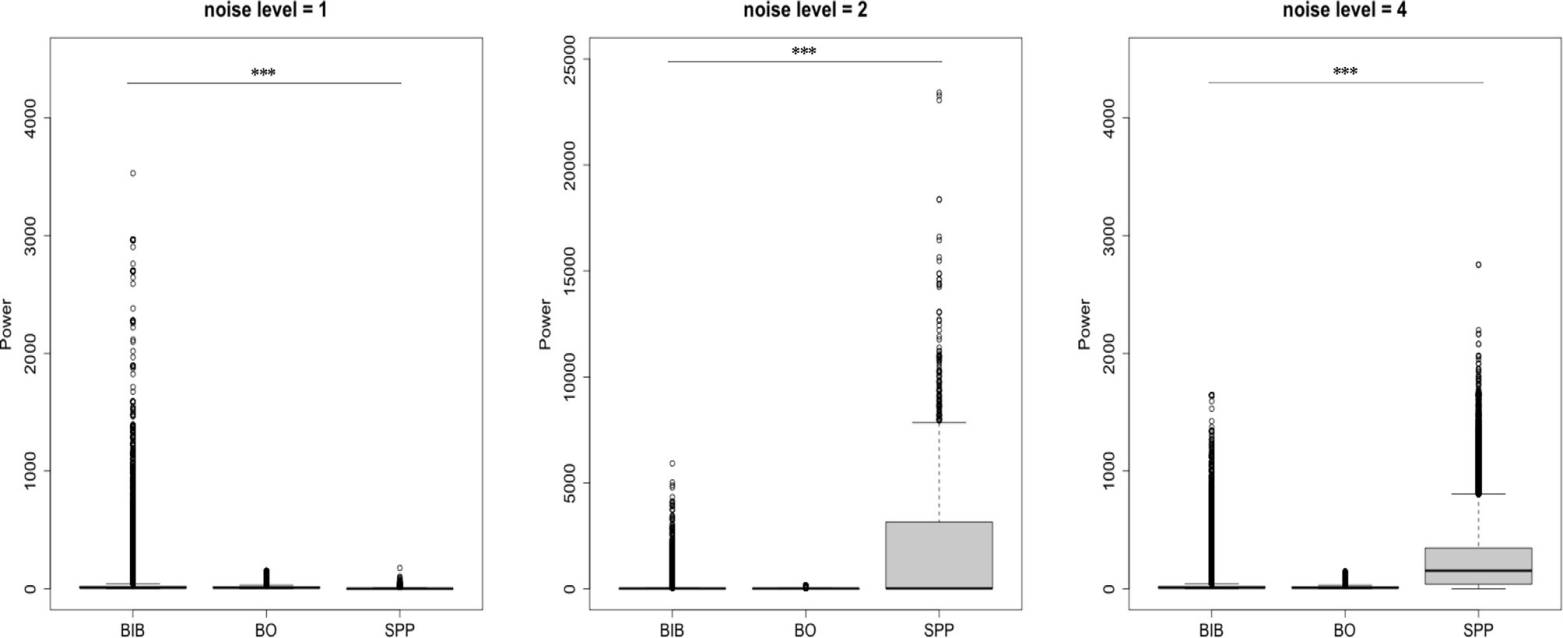
Comparisons among the step lengths in the BIB, BO and SPP models under various levels of noise. Under each level, the models are significantly different from each other.

First, we conducted a statistical test, the Kruskal-Wallis rank sum test, to determine whether there was a significant difference among the distributions of the step length in the SPP, BO and BIB models. Since the distribution of the step length in the BIB swarm is far from a normal distribution, the test is nonparametric, and we adopted the Kruskal-Wallis rank sum test. It was found that all differences among the distributions of the step length in the SPP, BO and BIB models were significant (p < 0.001, all are smaller than 2.2e-16). Figure 11 shows the comparison of the mean and variance among the BIB, BO and simple SPP models under various levels of noise. Noise levels 1, 2 and 4 imply that *ε* is 0.1, 0.2 and 0.4, respectively.

For the trajectories from the central area in an open space, the distribution of the step length is estimated. As mentioned before, the walk bending at a smaller angle than 2*π*/9 is regarded as a straight walk. If the step length and its frequency are represented by *D* and *f*(*D*), respectively, the power law distribution is expressed as:

(24) $$f\left(D\right)\propto D^{-\mu }$$

and if 1≤μ≤3, it is called the Lévy walk. Recent studies have demonstrated that animal walks are approximated as truncated power law distributions [46], [47] such as:

(25) $$f\left(D\right)=D^{-\mu }e^{\mathrm{aD}}$$

Regarding the step length distributions of the simple SPP, BO and BIB, we test whether the distribution is approximated by the truncated power law distribution or the exponential distribution with the Akaike Information Criterion (AIC) [75], [76], [77], [78]. Truncated power law distribution is frequently used for data fitting in Lévy walk analysis, while there is a trade-off between efficiency of fitting and completeness in data fitting [79], [80], [81].

Two probability density functions are given, one for the truncated power law distribution, $f\left(x\right)=\left(\mu -1\right)/\left(x_{min}^{1-\mu }-x_{max}^{1-\mu }\right)x^{-\mu }$, and the other for the exponential distribution, $f\left(x\right)=\lambda exp\left(-\lambda \left(x-x_{min}\right)\right)$, where $x_{min}$ is determined by using Kolmogorov-Smirnov statistics and $x_{max}$ is defined as the maximum value of the data [82]. After best-fit exponents for the truncated power law (*μ*) and the exponential distribution (*λ*) and log-likelihood are calculated, the AIC and Akaike weight for both models are calculated. Finally, the better-fitting model is determined based on the Akaike weight.

**Figure 12:**
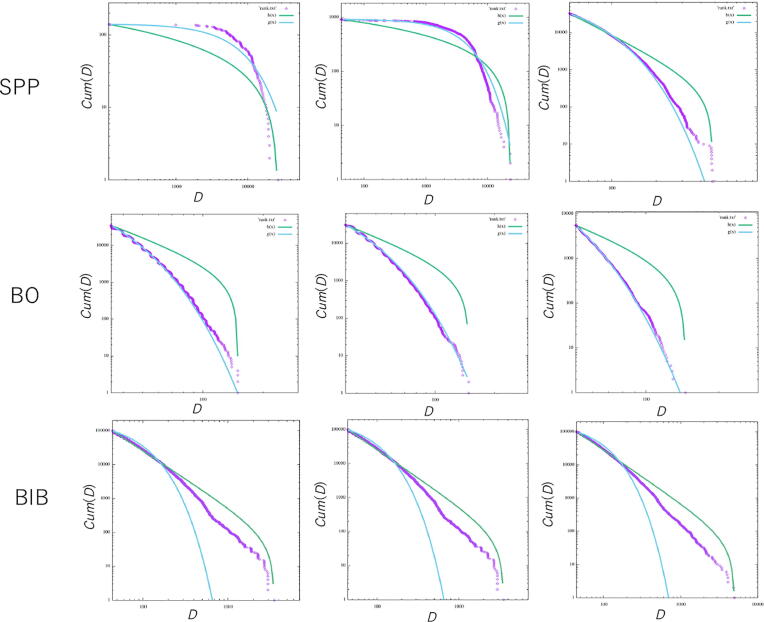
Cumulative frequency distribution of the step length generated by the simple SPP (above), the SPP with only Bayesian inference (BO; middle) and the SPP with Bayesian and inverse Bayesian inference (BIB; below) plotted against the normalized step length (purple squares). The data are approximated by two distributions, i.e., the exponential (blue) and truncated power law (green) distributions. From left to right, the perturbation *ε* is set to 0.1, 0.2, and 0.4.

Figure 12 shows the cumulative frequency distribution for the step length. We calculated the Akaike weight for the truncated power law distribution, 0.0≤wpl≤1.0, where the larger wpl is, the higher the likelihood of the truncated power law distribution is. In contrast, 1-wpl represents the likelihood for the exponential distribution. The left, middle and right columns represent the conditions with *ε* equal to 0.1, 0.2, 0.4, respectively. In the case of the simple SPP (above panel in Figure 12), for *ε* = 0.1, *μ*=1.00, *λ*=0.00, wpl=0.00, for *ε* = 0.2, *μ*=1.00, *λ*=0.00, wpl=0.00, and for *ε* = 0.4, *μ*=3.00, *λ*=0.03, wpl=0.00, respectively. This implies that the frequency distribution of the SPP best fits to an exponential distribution. In the case of the BO, for *ε* = 0.1, *μ*=3.00, *λ*=0.09, wpl=0.00, for *ε* = 0.2, *μ*=3.00, *λ*=0.08, wpl=0.00, and for *ε* = 0.4, *μ*=3.00, *λ*=0.10, wpl=0.00. This also implies that the frequency distribution of the BO model best fits to an exponential distribution. In contrast, the BIB reveals different types of distributions. In the case of the BIB, for *ε* = 0.1, *μ*=2.65, *λ*=0.02, wpl=1.00, for *ε* = 0.2, *μ*=2.59, *λ*=0.02, wpl=1.00, and for *ε* = 0.4, *μ*=2.72, *λ*=0.02, wpl=1.00. Thus, the step length distribution generated by the BIB model strictly indicates a power law distribution, especially Lévy walk, and critical behavior.

**Figure 13:**
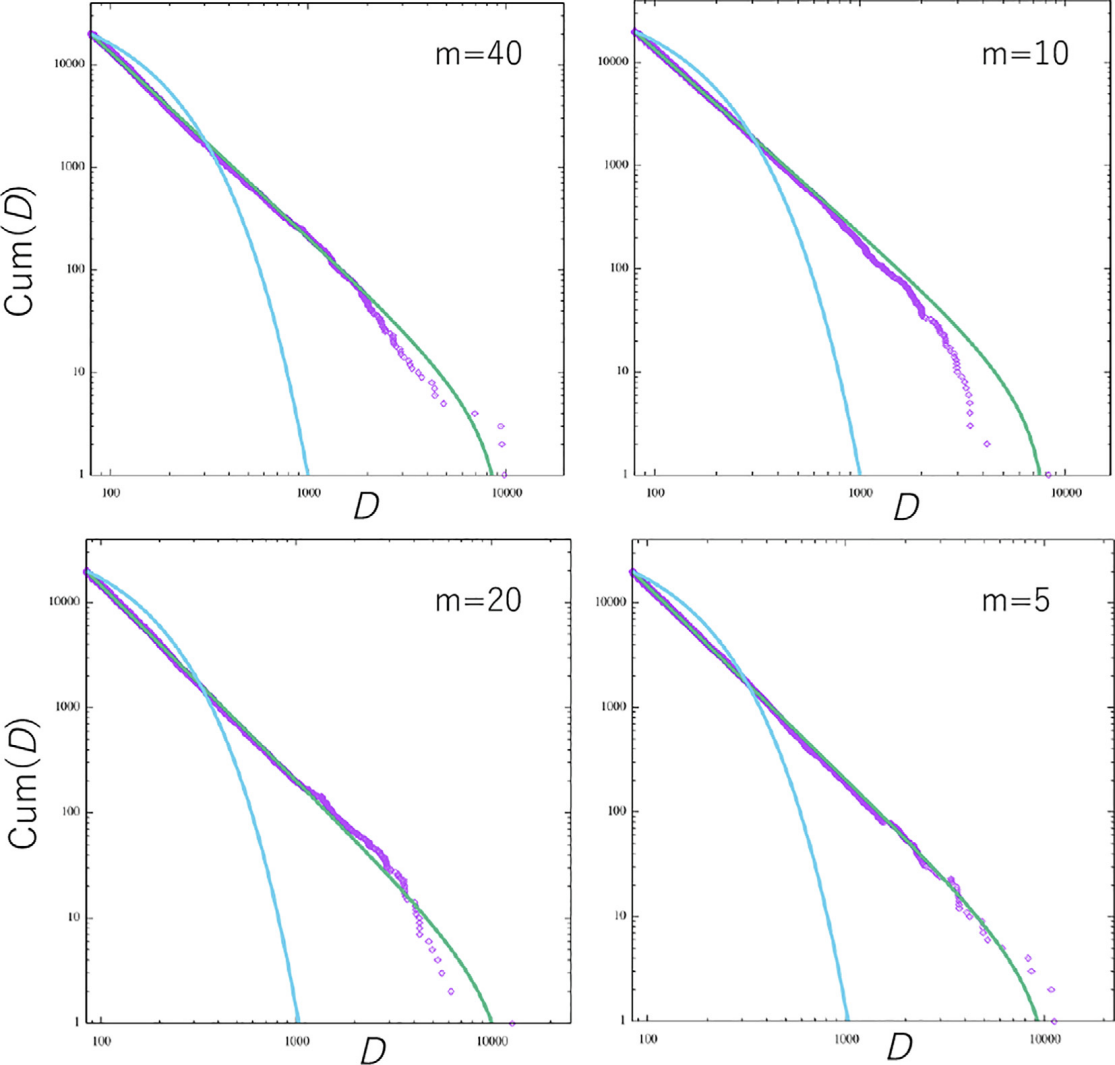
Cumulative frequency distributions of the step length for various window sizes of the BIB inference. The data are approximated by two distributions, i.e., the exponential (blue) and truncated power law (green) distributions. The perturbation *ε* is set to 0.001.

We checked whether the window size of the BIB inference (m) influenced the results of the step length distribution. We found that the window size did not influence the power law distribution, as shown in Figure 13. The exponent for the truncated (*μ*) and exponential (*λ*) power law for various windows were obtained by the following: for m=40, *μ* =2.81 and *λ* = 0.011; for m=20, *μ* =2.85 and *λ* = 0.010; for m=10, *μ* =2.78 and *λ* = 0.011; and for m=5, *μ* =2.8 and 5*λ* = 0.010. The Akaike weights, wpl, for all window sizes were 1.0, and then all step length distributions could be fit to a truncated power law distribution.

Finally, it is confirmed that the complex patterns consisting of tornado, splash and translation behaviors, generated by the BIB, imply critical behaviors. In other words, the interaction between data and hypotheses in Bayesian inference can self-organize its critical behavior. This kind of criticality is intrinsically different from the criticality of the phase transition. As mentioned before, a phase transition occurs in the SPP. With respect to the step length distribution, one cannot determine a power law distribution, and there are signs of critical phenomena only encountered at the critical point. In contrast, the criticality in the BIB is not observed in narrow critical regions. Over a wide range of perturbations (*ε*) and window size (m), power law distribution can be found. In this sense, the criticality is ubiquitously found and could be called a universal criticality.

## 4 Discussion and Conclusion

First, we consider the problem of artificial and natural design to be realized in open environments. Since the process of genome editing was first proposed and developed, it has become possible to design an artificial genome specific to a particularly concrete function [83], [84], [85]. Artificial design involves the hard problem of which optimal design based on the above concrete function must be achieved in an open and real environment. There are two reasons why the artificial and natural design is challenging. The first is how to overcome the trade-off between the behavior under open conditions and the behavior specific to a certain function [1], [2]. The second reason is, if one could assign a solution beyond the above trade-off, how could the solution be effectively obtained [3], [4]. These two reasons are mutually related to each other. Herein, we proposed a kind of solution to this problem.

The above design faces the trade-off between open conditions and definite functions, and the trade-off can be replaced by the trade-off between universality and efficiency in computation and by the trade-off between exploration and exploitation. In this sense, the design to be achieved results in the coexistence of both exploration and exploitation beyond the trade-off. In a swarm model, called the SPP, the trade-off between exploration and exploitation is expressed as the phase transition between order and chaos. Thus, it is expected that universal and efficient design can be realized at the critical point or the edge of chaos in the SPP parameter (perturbation) space.

Although determining the critical point as the design solution requires parameter fine tuning, there have been attempts to autonomously identify the critical point, that is called metaheuristics. One of these attempts is the self-organizing criticality (SOC). While the SOC has been applied to determining the criticality in the SPP, previous attempts have failed to identify the critical behavior without ad hoc global knowledge such as a fitness function. The metaheuristics for the swarm model based on SOC require global knowledge such as a fitness function [63], [64]. As such, the idea of the SOC is not autonomously easy beyond parameter tuning. While there are many swarm-based optimization techniques [33], [34], [35], [36], they are not related to the critical state.

Our proposal based on the interaction between data and hypotheses in Bayesian inference easily achieves critical behavior. However, this is different from the idea of the SOC with respect to two points. First, while the SOC is a way to choose the optimal solution from among possible states, our proposal does not select a limited state from many possible states. Instead of choosing, our proposal turns most states into critical states. In particular, metaheuristics are not introduced as fitness is added to the phase transition but instead the phase transition itself is modified. Therefore, critical behavior is not achieved only at the edge of chaos but is achieved anywhere in the parameter space. Second, our proposal never requires fitness knowledge or metaheuristics. The agent of the SPP never sees all agents in a space, and each agent makes decisions based on Bayesian and inverse Bayesian (BIB) inference, which is a task based not on global knowledge but on local dynamic knowledge.

Bayesian inference implements information contraction since it replaces the probability with the conditional probability. The agent using Bayesian inference reduces the world to that experienced by itself. Thus, this occurs in the simple optimization framework, and Bayesian inference contributes to rapidly reaching the optimal solution. Inverse Bayesian inference implements information extension since it replaces the conditional probability with the probability. In other words, the agent perpetually perceives the real world (data) outside its own cognitive world (hypotheses). Because the combination of information contraction and extension is independent of the knowledge of fitness, it could be applied to various problems.

Although our proposal is based on agents implementing an inference system, it never assumes that the genome has the ability to make decisions even if our proposal is applied to, for instance, genome editing. The optimal design of a network or traveling salesman problem can be resolved by ant agents making decisions with the probability or pheromones. It is never implied that the network itself has the ability to make decisions. Bayesian and inverse Bayesian inference denote that the probability is not globally given but is temporally and locally defined. Balancing information contraction and extension in Bayesian and inverse Bayesian inference leads to the universal criticality.
